# Adherence to preventive measures after SARS-CoV-2 vaccination and after awareness of antibody response in kidney transplant recipients in the Netherlands: a nationwide questionnaire study

**DOI:** 10.1016/j.eclinm.2023.102103

**Published:** 2023-07-20

**Authors:** Sophie C. Frölke, Pim Bouwmans, A. Lianne Messchendorp, Johanna P.M. Vervoort, Alferso C. Abrahams, Aiko P.J. de Vries, Pythia T. Nieuwkerk, Marc H. Hemmelder, Ron T. Gansevoort, Luuk B. Hilbrands, Marlies E.J. Reinders, Jan-Stephan F. Sanders, Frederike J. Bemelman, Suzanne E. Geerlings, RECOVAC Collaborators, C. Imhof, C. Idzinga, C. Siegert, C.C. Baan, C.J.A.M. Konings, C. van Kessel, D. van Baarle, D.A. Diavatopoulos, D. Standaar, E. ten Hoope, E. Til, E.B.M. Remmerswaal, F. van der Klis, H.R. Fritsen, I. Stijnman, J.N. Brinkman, J. Cheng, L. den Biggelaar, M. ten Dam, M. Steenhuis, M. Zwerink, M.H.J. Braks, M. Willems, M.L. Kho, N. Rots, P. Vart, R.G. van der Molen, R.M.A. van den Dorpel, R.S.R.K. Malaha, R.C.G. ter Meulen, T. Rispens, T. Steenvoorden, T. de Ronde, V.J.P. Peters, W.S. Konijn, W.M.T. Janssen, W.J. Bos, Y.M.R. Adema, Y. Vegting

**Affiliations:** a Amsterdam UMC Location University of Amsterdam, Renal Transplant Unit, Meibergdreef 9, Amsterdam, the Netherlands; b Amsterdam Institute for Infection and Immunity, Infectious Diseases, Amsterdam, the Netherlands; c Division of Nephrology, Department of Internal Medicine, Maastricht University Medical Centre, Maastricht, the Netherlands; d CARIM School for Cardiovascular Diseases, Maastricht University, Maastricht, the Netherlands; e Division of Nephrology, Department of Internal Medicine, University of Groningen, University Medical Centre Groningen, Groningen, the Netherlands; f Dutch Kidney Patient Association (NVN), Bussum, the Netherlands; g Global Health Unit, Department of Health Sciences, University of Groningen, University Medical Centre Groningen, Groningen, the Netherlands; h Department of Nephrology and Hypertension, University Medical Centre Utrecht, Utrecht, the Netherlands; i Department of Medicine, Division of Nephrology, Leiden University Medical Centre, Leiden, the Netherlands; j Leiden Transplant Centre, Leiden, the Netherlands; k Amsterdam UMC Location University of Amsterdam, Medical Psychology, Meibergdreef 9, Amsterdam, the Netherlands; l Amsterdam Public Health, Quality of Care, Amsterdam, the Netherlands; m Department of Nephrology, Radboud University Medical Centre, Radboud Institute for Health Sciences, Nijmegen, the Netherlands; n Department of Internal Medicine, Nephrology and Transplantation, Erasmus MC Transplant Institute, Erasmus University Medical Centre, Rotterdam, the Netherlands; o Amsterdam UMC Location University of Amsterdam, Infectious Diseases, Meibergdreef 9, Amsterdam, the Netherlands

**Keywords:** Kidney transplantation, SARS-CoV-2, Vaccination, Behavior, Adherence

## Abstract

**Background:**

Kidney transplant recipients (KTRs) were advised to tightly adhere to government recommendations to curb the spread of severe acute respiratory syndrome coronavirus-2 (SARS-CoV-2) because of a high risk of morbidity and mortality and decreased immunogenicity after vaccination. The aim of this study was to analyse the change in adherence to preventive measures after vaccination and awareness of antibody response, and to evaluate its effectiveness.

**Methods:**

In this large-scale, national questionnaire study, questionnaires were sent to 3531 KTRs enrolled in the Dutch RECOVAC studies, retrospectively asking for adherence to nine preventive measures on a 5-point Likert scale before and after SARS-CoV-2 vaccination and after awareness of antibody response. Blood samples were collected 28 days after the second vaccination. Antibody response was categorised as non-responder (≤50 BAU/mL), low-responder (>50 ≤ 300 BAU/mL) or high-responder (>300 BAU/mL), and shared with participants as a correlate of protection. Participants of whom demographics on sex and age, blood samples and completed questionnaires were available, were included. Our study took place between February 2021 and January 2022. The primary outcome of adherence before and after vaccination was assessed between August and October 2021 and compared via the Wilcoxon signed rank sum test. Logistic regression analysis was performed to estimate the association between antibody response and non-adherence, and adherence on acquiring SARS-CoV-2 infection. This study is registered at ClinicalTrials.gov (NCT04841785).

**Findings:**

In 2939 KTRs (83%) who completed the first questionnaire on adherence to preventive measures, adherence was higher before than after vaccination (4.56, IQR 4.11–4.78 and 4.22, IQR 3.67–4.67, p < 0.001). Adherence after awareness of antibody response was analysed in 2399 KTRs (82%) of whom also blood samples were available, containing 949 non-responders, 500 low-responders and 950 high-responders. Compared to non-responders, low- and high-responders reported higher non-adherence. Higher adherence was associated with lower infection rates before and after vaccination (OR 0.67 [0.51–0.91], p = 0.008 and OR 0.48 [0.28–0.86], p = 0.010).

**Interpretation:**

Adherence decreased after SARS-CoV-2 vaccination and in KTRs who were aware of a subsequent antibody response compared with those without. Preventive measures in this vulnerable group seem to be effective, regardless of vaccination status. This study starts a debate on sharing antibody results with the patient and future studies should elucidate whether decreased adherence in antibody responders is justified, also in view of future pandemics.

**Funding:**

The Netherlands Organization for Health Research and Development and the 10.13039/501100002997Dutch Kidney Foundation.

**Research in context**

**Evidence before this study**

We searched PubMed for studies about adherence to government recommendations to prevent severe acute respiratory syndrome coronavirus-2 (SARS-CoV-2) infection in immunocompromised patient groups, published until January 2023 using the following terms “Kidney Transplantation” OR “Immunocompromised Host”, AND “SARS-CoV-2” OR “COVID-19”, AND “Behavior” OR “Protective Factors” OR “Patient Compliance” OR “(Guideline) Adherence”. Three survey studies exist, one showing tight adherence to government recommendations in kidney transplant recipients resulting in no reported infections and two in other immunocompromised groups compared with a control group. No longitudinal papers exist that report the association between behavioral changes in these patients and SARS-CoV-2 incidence. Keeping in mind, on one hand, the high risk of morbidity and mortality of coronavirus disease 2019 (COVID-19) in kidney transplant recipients and on the other hand the risk of mental health problems as a result of social isolation, proof of the effectiveness of this adherence is needed.

**Added value of this study**

In this large-scale, national questionnaire study, we retrospectively asked kidney transplant recipients to self-report their adherence to preventive measures before SARS-CoV-2 vaccination, after vaccination, and after awareness of antibody response. We found lower adherence after vaccination in all kidney transplant recipients, despite the higher risk of severe COVID-19 in kidney transplant recipients with limited antibody formation. Subsequently, we observed an independent dose-response relationship between awareness of higher antibody response and higher non-adherence to nearly all preventive measures. Most importantly, we showed that preventive measures in this vulnerable group are effective in the prevention of SARS-CoV-2 infection, independent of vaccination status. To our knowledge, this is the first report of such findings.

**Implications of all the available evidence**

This study starts a debate on sharing antibody results with the patient. In this context, the goal of measuring antibody levels in clinical practice is to give kidney transplant recipients more freedom in terms of preventive measures as social interaction is essential to every aspect of their health. However, this situation is a challenging one, given that it is still unknown what antibody level is protective against COVID-19 in kidney transplant recipients. Future studies should elucidate whether decreased adherence in antibody responders is justified, also in view of future pandemics.

## Introduction

At the start of the coronavirus disease 2019 (COVID-19) pandemic in December 2019, kidney transplant recipients (KTRs) were at high risk of complications in case of severe acute respiratory syndrome coronavirus-2 (SARS-CoV-2) infection. In 2020, the COVID-19-associated mortality risk was reported to be 3–4 times higher in transplant recipients than in the general population.^1^ Therefore, the availability of effective and safe SARS-CoV-2 vaccines was of great importance for this vulnerable group. When SARS-CoV-2 vaccination became available in 2021, it was yet uncertain whether these vaccines were equally effective as in the general population. In the general population, seroconversion was observed in nearly all participants in the phase 2/3 SARS-CoV-2 messenger ribonucleic acid (mRNA) vaccination trials.^2^^,^^3^ Unfortunately, immunocompromised patients were not included in these studies. However, because of their observed susceptibility for severe disease, KTRs were prioritised in the vaccination program in the Netherlands.^4^

First results of the immune response after two SARS-CoV-2 vaccinations in KTRs showed a decreased seroconversion rate ranging from 30% to 57%.^5^, ^6^, ^7^, ^8^, ^9^, ^10^ Given the association between antibody response and protection against severe COVID-19,^11^^,^^12^ a majority of KTRs was therefore still at risk of severe disease.^1^^,^^11^^,^^13^^,^^14^ The most efficient way to prevent virus transmission, was to strictly follow the recommendations issued by the Dutch government for social isolation and preventive measures against COVID-19 as published on the website of the National Institute for Public Health and the Environment (RIVM).^15^ The behavioral changes after SARS-CoV-2 vaccination and awareness of antibody response, however, have not yet been studied in KTRs.

The first aim of this study was to describe the change in self-reported adherence to preventive measures after SARS-CoV-2 vaccination. Secondly, we compared adherence in groups with different antibody responses after vaccination. Thirdly, the effectiveness of these government recommendations in KTRs were evaluated. We hypothesised that KTRs would be less adherent to protective measures after SARS-CoV-2 vaccination, and after awareness of an antibody response. Protective measures were expected to be effective against SARS-CoV-2 infection.

## Methods

### Study population

We invited all 12.159 KTRs in the Netherlands to participate in the Long-term Efficacy and Safety of SARS-CoV-2 vaccination (LESS CoV-2) prospective cohort study, conducted by the Dutch REnal patients COVID-19 VACcination (RECOVAC) consortium. The design has previously been published in detail elsewhere (ClinicalTrials.gov Identifier: NCT04841785).^16^^,^^17^ KTRs were invited via the national COVID-19 vaccination program. These patients were prioritised for vaccination, which was facilitated by their treating hospitals. Through a joint collaboration with all hospitals in the Netherlands we informed KTRs about this study via a national campaign including information on websites for KTRs, direct mailing, flyers and posters. As the vaccination rate amongst KTRs in the Netherlands was very high, almost all patients in the Netherlands had the possibility to apply for this study either by QR-code or returning the flyer including their contact information. Participants received their second SARS-CoV-2 vaccination between February and July 2021. In the Netherlands, initial vaccination could be carried out with the mRNA vaccines mRNA-1273 (Moderna) or BNT162b2 (Pfizer-BioNTech), or the vector vaccines ChAdOx nCov19 (AstraZeneca) or Ad26.CoV2.S (Janssen). From May till August 2021 blood samples were obtained by use of a home-based finger prick kit (Sanquin, Amsterdam, The Netherlands), around 28 days after the second SARS-CoV-2 vaccination, in which anti-SARS-CoV-2 receptor-binding domain (RBD) immunoglobulin G (IgG) antibody levels were analysed. For our first analysis of the change in adherence to preventive measures after SARS-CoV-2 vaccination, participants of whom demographics on sex and age were available and who had completed the first of two questionnaires on adherence were included. For the second analysis of adherence to preventive measures after awareness of antibody response, participants were excluded if no second questionnaire or blood sample was available.

### Questionnaires and antibody level communication letter

All KTRs (N = 3531) in the LESS CoV-2 study, retrospectively reported their level of adherence to preventive measures in three different periods: before vaccination, after vaccination and after receiving the results of their antibody response. These questionnaires were received either by e-mail or by regular mail, whichever was preferred. The first questionnaire was obtained between August and October 2021 in which participants reported simultaneously on the period before and after the second SARS-CoV-2 vaccination. After completing this questionnaire, participants had insight into their individual antibody level after vaccination through a separate communication letter. Participants were informed whether they were considered as a non-responder (≤50 binding antibody units (BAU)/mL), low-responder (>50 ≤ 300 BAU/mL) or high-responder (>300 BAU/mL) against the wild-type SARS-CoV-2 variant, and what this might indicate for their level of protection. This indication was described as follows: less than 50 BAU/mL means no antibodies were developed after vaccination and the risk of (severe) COVID-19 is the same as before vaccination. Between 50 and 300 BAU/mL means less antibodies were developed compared to healthy controls and the risk of (severe) COVID-19 is smaller than before vaccination, but remains high. More than 300 BAU/mL means comparable antibody levels to healthy controls, but just as in the latter population, the risk of (severe) COVID-19 remains.

The second questionnaire was obtained between December 2021 and January 2022 for the period after participants received their antibody response. Participants also received two self-report surveys, approximately 1 month and 6 months after the second SARS-CoV-2 vaccination, containing questions about previous SARS-CoV-2 infection (yes/no) and about general health. To minimise the burden, we sent out one single remainder to individuals who did not respond, either by e-mail or regular mail. An overview of the timing of all study events in perspective of the COVID-19 pandemic including infection and hospitalisation rate and circulating variants, is shown in Supplementary Figure S1. Details on primary kidney disease and transplant characteristics were collected from the Dutch Organ Transplant Registry (NOTR), a national registry for KTRs.

The nine preventive measures were described as follows. *‘Keep a distance of 1.5 meters from another’*, *‘Wearing a face mask if mandatory or if social distancing is not possible’*, *‘Washing hands’*, *‘Avoiding supermarket or other shops’*, *‘Avoiding public transport’*, *‘Avoiding crowded gatherings’, ‘Limit visitors or visits’*, *‘Working from home’* and *‘Avoiding travel abroad’*. Adherence to preventive measures was reported on a 5-point Likert scale, ranging from never (Likert score 1) to always (Likert score 5). The answer options ‘Don’t know’ (Likert score 6) and ‘Not applicable’ (Likert score 7) were also included. We used self-designed questionnaires constructed by a project group consisting of a project leader (M. H. H.), two researchers (A. L. M. and P. B.) and five kidney patient organisations’ representatives of the Dutch Kidney Patient Association (NVN), which included kidney patients, experienced in quality of care in order to maximise content validity of the questionnaires.

### Antibody measurement

Antibody levels were measured in serum by using the Sanquin anti-SARS-CoV-2 RBD IgG enzyme linked immunoassay (ELISA), approximately 28 days after the second SARS-CoV-2 vaccination. Previous exploratory analysis of the association between SARS-CoV-2-binding antibody concentration and neutralising antibody titre enabled us to define a cutoff level of 300 BAU/mL to categorise responders into high-responders (>300 BAU/mL)^18^ and low-responders (>50 BAU/mL but ≤300 BAU/mL).^17^^,^^19^ This cut-off level was based on a plaque reduction neutralisation test titre of 40 that we considered as minimally protective.

### Ethics

All participants of the LESS CoV-2 study were aged 18 years or older and provided informed consent. The Medical Research Ethics Committee of the University Medical Centre Groningen has granted approval to carry out this study (EudraCT nr.: 2021-001520-18). The study is conducted according to the principles of the Declaration of Helsinki (64th WMA General Assembly, Fortaleza, Brazil, October 2013) and in accordance with the Medical Research Involving Human Subjects Act (WMO). All results are presented according to the Strengthening the Reporting of Observational Studies in Epidemiology (STROBE) Initiative.^20^

### Statistical analysis

Distribution of variables was assessed by the eye-ball test for histograms and mean minus 2∗SD should equal realistic results for the variable. Variables are presented as mean ± standard deviation (SD) if normally distributed, or as median and interquartile range (IQR) in case of non-normal distribution. p-values were calculated using independent sample t test for normally distributed continuous variables, Mann–Whitney U test in case of non-normally distributed continuous variables and chi-square test in case of categorical variables. For our regression analyses, we selected potential confounders based on clinical knowledge and/or existing literature. We considered the following variables as potential confounders: sex, European descent, age, Body Mass Index (BMI), primary renal diagnosis, number of comorbidities, estimated glomerular filtration rate (eGFR), first kidney transplant yes/no and time after transplantation, vaccine type, number of immunosuppressive agents and previous SARS-CoV-2 infection. Second, we introduced each potential confounder to the univariable regression model with the variable of interest. If this changed the regression coefficient of the variable of interest with more than 10%, it was selected as confounder for the final association model.

First, adherence to preventive measures before SARS-CoV-2 vaccination and after SARS-CoV-2 vaccination was compared by using the Wilcoxon signed rank sum test. For this analysis, we calculated an average adherence score over all preventive measures by adding up all Likert scores as continuous variables and divide it by nine, the total number of preventive measures. The average adherence score was considered a continuous variable. We estimated whether previous SARS-CoV-2 infection was associated with the log-transformed adherence after vaccination, by using linear regression analysis, in which we corrected for reported adherence before vaccination.

In the second analysis, adherence to preventive measures was treated according to the Likert scale, as an ordinal variable. We estimated whether antibody response group was associated with adherence to each of the preventive measures by univariable ordinal logistic regression, taking non-responders as the reference group. We inverted adherence to non-adherence by taking 1/OR to generate odds ratios greater than 1 to improve interpretability. Thus, the higher non-adherence, the less tightly participants adhered to preventive measures compared to the reference group. Next, we performed multivariable ordinal logistic regression analysis. The selected confounding variables were clustered in three different models. Model 0 represents the crude analysis. In model 1, we introduced age, followed by previous SARS-CoV-2 infection in model 2. Previous SARS-CoV-2 infection was defined as infection before blood withdrawal for antibody level measurement. In additional sensitivity analysis, we took low-responders as the reference group, which we performed in exactly the same manner.

Lastly, we assessed the association between adherence to preventive measures before and after SARS-CoV-2 vaccination on acquiring SARS-CoV-2 infection by logistic regression. Observations of infection after the second vaccination were censored at the time participants received the results of their antibody response or of the third SARS-CoV-2 vaccination, whichever came first (right censoring).

The answer options ‘Don’t know’ (Likert score 6) and ‘Not applicable’ (Likert score 7) were handled as missing responses. We performed complete case analysis. Data was assumed to be missing at random. Statistical analysis was performed using R version 4.0.3. A two-sided p-value of <0.05 was considered as statistically significant.

### Role of the funding source

The funders of the study had no role in study design, data collection, data analysis, data interpretation, or writing of the report.

## Results

**Fig. 1:**
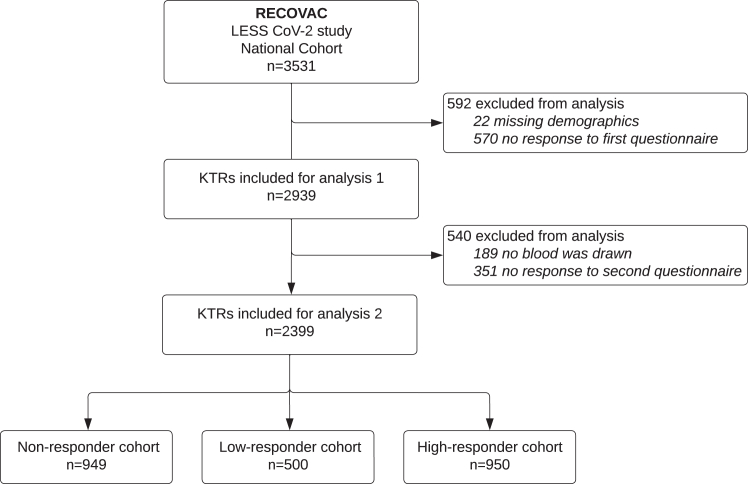
Flow diagram of study inclusion for analysis 1 (change in adherence after severe acute respiratory syndrome coronavirus-2 vaccination) and analysis 2 (adherence after awareness of antibody response). KTRs, kidney transplant recipients.

In total, 2939 eligible KTRs were included (83%) for the first study analysis of the change in adherence after SARS-CoV-2 vaccination (Fig. 1). A number of 2399 patients remained (82%) for the second study analysis to analyse the association between awareness of antibody response and adherence. Table 1 shows the descriptive characteristics of these KTRs according to the three levels of antibody response as reported to the patients (see Supplementary Table S1 for percentage of variable missings). Patients in the high-responder group (N = 950) were younger (mean age 56.6 years, SD 12.8) compared to the low-responder group (N = 500) (mean age 59.8 years, SD 11.6) and non-responder group (N = 949) (mean age 61.5 years, SD 11.4). Most of participants in our cohort were male (57%), and sex distribution was comparable between groups. The vast majority of participants received the mRNA-1273 vaccine. The antibody concentration was measured on average 34.3 days (SD 13.7) after the second vaccination. The median antibody titre was 7 BAU/mL (IQR 1–19) in the non-responder, 122 BAU/mL (IQR 79–177) in the low-responder and 1692 BAU/mL (IQR 741–3361) in the high-responder group. A total number of 592 patients were excluded from the first study analysis (Supplementary Table S2). For the second study analysis, we subsequently excluded 540 patients, of which from 83 patients insufficient volume was received for antibody measurement (Supplementary Table S3). The excluded patients were younger, received less immunosuppressive agents and had less comorbidities. Additionally, we provided an approximation of characteristics of the excluded national cohort of KTRs who were invited via the national vaccination program, but did not agree to participate and therefore were not enrolled in the LESS CoV-2 study (please see Supplementary Table S4 for more information). Compared to the LESS CoV-2 cohort included in the first analysis, the patients in the national exclusion cohort were also younger and received less immunosuppressive agents.

**Table 1:** Descriptive statistics of the three groups.

	Non-responder group Total (N = 949)	Low-responder group Total (N = 500)	High-responder group Total (N = 950)
Sex, n (%)			
Male	520 (54.8)	298 (59.6)	551 (58.0)
Female	429 (45.2)	202 (40.4)	399 (42.0)
European descent, n (%)^a^	843 (93.0)	453 (93.2)	843 (91.2)
Age, y	61.5 (11.4)	59.8 (11.6)	56.6 (12.8)
BMI, kg/m^2^^a^	25.9 (4.4)	25.9 (4.1)	26.2 (4.3)
Current smoking, n (%)^a^			
Yes	57 (6.1)	36 (7.3)	43 (4.6)
Past	476 (51.1)	220 (44.5)	429 (45.8)
Never	399 (42.8)	238 (48.2)	465 (49.6)
Current alcohol consumption, n (%)^a^			
Daily	547 (58.8)	268 (54.3)	511 (54.7)
Less than daily	297 (31.9)	175 (35.4)	358 (38.3)
Never	87 (9.3)	51 (10.3)	66 (7.1)
Primary renal diagnosis, n (%)^a^			
Congenital/hereditary disease	22 (2.8)	14 (3.3)	37 (4.9)
Cystic kidney disease	136 (17.5)	67 (15.9)	145 (19.4)
Diabetic kidney disease	67 (8.6)	24 (5.7)	36 (4.8)
Glomerulonephritis	145 (18.6)	93 (22.1)	148 (19.8)
Interstitial nephritis/pyelonephritis/drug induced nefropathy/urolithiasis	47 (6.0)	34 (8.1)	60 (8.0)
Renal vascular disease	64 (8.2)	34 (8.1)	48 (6.4)
Other multisystemic disease	43 (5.5)	15 (3.6)	45 (6.0)
Other	231 (29.7)	112 (26.6)	178 (23.8)
Unknown	24 (3.1)	28 (6.7)	52 (6.9)
No. of comorbidities, n (%)			
None	93 (9.8)	59 (11.8)	138 (14.5)
1	422 (44.5)	255 (51.0)	494 (52.0)
2	280 (29.5)	116 (23.2)	210 (22.1)
≥3	154 (16.2)	70 (14.0)	108 (11.4)
Comorbidities, n (%)			
Diabetes mellitus	279 (29.4)	104 (20.8)	209 (22.0)
Chronic lung disease	92 (9.7)	33 (6.6)	45 (4.7)
Heart failure	66 (7.0)	29 (5.8)	52 (5.5)
Hypertension	799 (84.2)	428 (85.6)	771 (81.2)
Coronary artery disease	134 (14.1)	63 (12.6)	84 (8.8)
Peripheral vascular disease	34 (3.6)	22 (4.4)	32 (3.4)
Malignancy	29 (3.1)	14 (2.8)	34 (3.6)
Stroke	63 (6.6)	27 (5.4)	36 (3.8)
Dementia	0 (0.0)	0 (0.0)	1 (0.1)
Liver cirrhosis	9 (0.9)	3 (0.6)	8 (0.8)
HIV/AIDS	3 (0.3)	0 (0.0)	3 (0.3)
eGFR, mL/min/1.73 m^2^^a^	49.0 (17.8)	52.9 (20.5)	51.4 (18.7)
Transplant characteristics^a^			
Transplantation type			
DBD	183 (24.6)	87 (20.9)	159 (21.7)
DCD	121 (16.2)	62 (14.9)	93 (12.7)
Living donation	441 (59.2)	267 (64.2)	481 (65.6)
First kidney transplant, n (%)	648 (87.0)	357 (85.8)	630 (85.9)
Time after transplantation, y	6.9 [3.7, 12.5]	7.6 [3.9, 13.9]	9.0 [4.9, 15.4]
Graft failure, n (%)	68 (9.1)	38 (9.1)	57 (7.8)
Vaccine type, n (%)^a^			
mRNA-1273	812 (87.1)	451 (91.5)	892 (95.2)
BNT162b2	79 (8.5)	27 (5.5)	34 (3.6)
ChAdOx-nCov19	40 (4.3)	15 (3.0)	11 (1.2)
Ad26.CoV2.S	1 (0.1)	0 (0.0)	0 (0.0)
No. of immunosuppressive agents, n (%)			
None	21 (2.2)	13 (2.6)	48 (5.1)
1	41 (4.3)	45 (9.0)	179 (18.8)
2	458 (48.3)	267 (53.4)	497 (52.3)
≥3	429 (45.2)	175 (35.0)	226 (23.8)
Immunosuppressive treatment, n (%)			
Steroids	653 (68.8)	361 (72.2)	601 (63.3)
Calcineurin inhibitor	768 (80.9)	375 (75.0)	729 (76.7)
MMF/MPA	750 (79.0)	283 (56.6)	289 (30.4)
Azathioprine	36 (3.8)	52 (10.4)	156 (16.4)
mTOR inhibitor	44 (4.6)	33 (6.6)	83 (8.7)
Antibody level, BAU/mL	7.0 [1.2, 19.4]	122.2 [78.7, 176.5]	1692.0 [740.5, 3360.5]

Abbreviations are: BMI, body mass index; HIV/AIDS, human immunodeficiency virus/acquired immunodeficiency syndrome; eGFR, estimated glomerular filtration rate; DBD, donation after brain death; DCD, donation after circulatory death; MMF/MPA, mycophenolate mofetil/mycophenolic acid; mTOR, mammalian target of rapamycin; BAU, binding antibody units.

a Due to missing values total numbers and values can vary.

### Association between SARS-CoV-2 vaccination and adherence

**Fig. 2:**
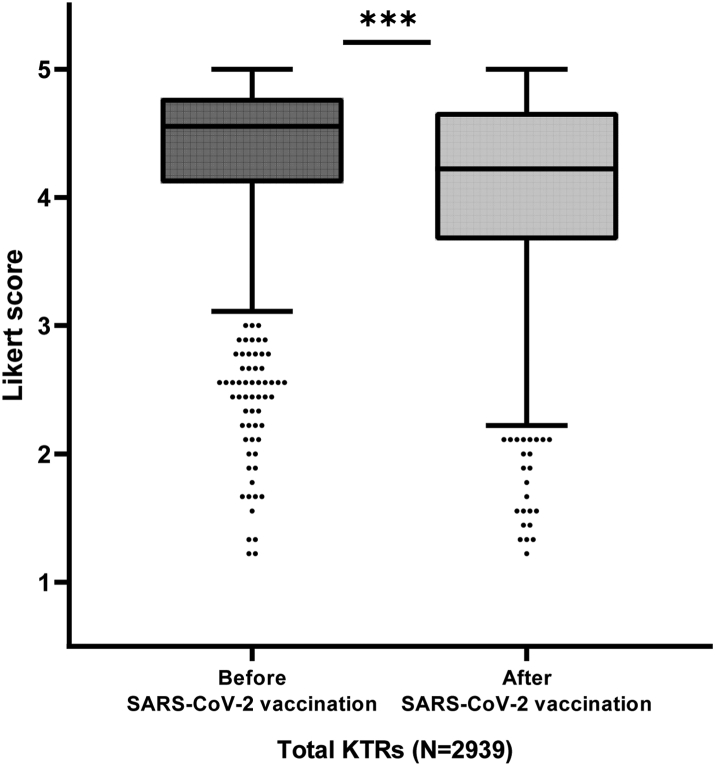
Boxplot for average adherence scores over all preventive measures before (4.56, interquartile range (IQR) 4.11–4.78) and after severe acute respiratory syndrome coronavirus-2 (SARS-CoV-2) vaccination (4.22, IQR 3.67–4.67) in kidney transplant recipients (KTRs). ∗∗∗Indicate a p-value of less than 0.001. Adherence to preventive measures was reported on a 5-point Likert scale, ranging from never (Likert score 1) to always (Likert score 5).

Overall, the average self-reported adherence to preventive measures was higher for the time period before vaccination (Likert score 4.56, IQR 4.11–4.78) than for the time period after vaccination (Likert score 4.22, IQR 3.67–4.67) (p < 0.001) (Fig. 2). This did not only account for the average adherence, but also for each of the specific preventive measures (Supplementary Figure S2, all p < 0.001). A total of 229 SARS-CoV-2 infections occurred during these two periods. Participants with previous SARS-CoV-2 infection did not differ in average adherence score after vaccination compared to participants without previous infection (exponentiated β: 0.99x, 95% CI 0.96–1.03, p = 0.664).

### Association between awareness of antibody response and adherence

**Fig. 3:**
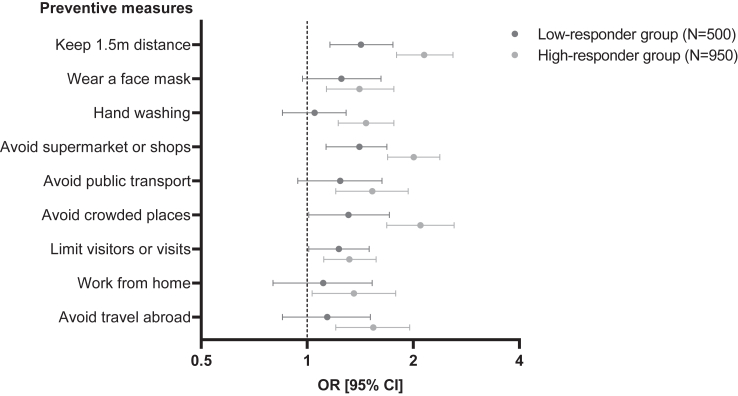
Forest plot for non-adherence to preventive measures after awareness of antibody response by group, corrected for the final association model (age and previous severe acute respiratory syndrome coronavirus-2 infection), taking the non-responder group as reference (N = 949). Please see Supplementary Table S5 for defined odds ratio (OR), confidence interval (CI) and p-value.

The average self-reported adherence to preventive measures after being informed of antibody response following vaccination on Likert scale was 4.2 (IQR 3.8–4.6) in non-responders, 4.1 (IQR 3.7–4.6) in low-responders and 3.9 (IQR 3.3–4.3) in high-responders (p < 0.001). High-responders had significantly higher non-adherence to each of the preventive measures after SARS-CoV-2 vaccination than non-responders, which also applied to most of the preventive measures in low-responders (Supplementary Table S5). In the final model, high-responders had higher non-adherence to all preventive measures compared to non-responders, independent of age and previous SARS-CoV-2 infection (N = 203). Compared to the non-responder group, low-responders had higher non-adherence to four out of nine preventive measures (keeping 1.5 m distance, avoiding supermarket or shops, avoiding crowded places and limiting visitors or visits) (Fig. 3). In additional sensitivity analysis comparing high-responders to low-responders, being a high-responder was associated with higher non-adherence to most of preventive measures (keeping 1.5 m distance, hand washing, avoiding supermarket or shops, avoiding crowded places and avoiding travel abroad) (Supplementary Table S6).

### Association of adherence with the risk of SARS-CoV-2 infection

In 2868 participants of whom data was available on SARS-CoV-2 infection before awareness of antibody response, a total of 202 SARS-CoV-2 infections occurred before vaccination and 27 occurred after vaccination. Of 27 infections 23 (85%) were confirmed by a PCR test. We found that a higher adherence to preventive measures was associated with a decreased likelihood of SARS-CoV-2 infection, both before (odds ratio (OR) 0.67, 95% confidence interval (CI) 0.51–0.91, p = 0.008) and after vaccination (OR 0.48, 95% CI 0.28–0.86, p = 0.010). The descriptive characteristics according to SARS-CoV-2 infection before and after vaccination are shown in Supplementary Tables S7 and S8, respectively. Due to the low number of infection events after vaccination we were statistically not powered to correct for any confounders in multivariable analysis. In the context of uniformity we decided not to correct for the period before vaccination.

## Discussion

To the best of our knowledge, we are the first to show that KTRs became less adherent to social isolation and other preventive measures after vaccination against COVID-19. KTRs who were aware of a subsequent antibody response were less likely to tightly adhere to preventive measures compared to those without an antibody response. Moreover, participants with higher adherence were at lower risk of SARS-CoV-2 infection, regardless of vaccination status.

Overall, KTRs perceived more freedom from preventive measures after SARS-CoV-2 vaccination, presumably because of an assumed development of SARS-CoV-2-specific antibodies and thus assumed protection against COVID-19. Since the majority of KTRs shows insufficient antibody response after two doses of mRNA vaccination,^5^, ^6^, ^7^, ^8^, ^9^, ^10^ the perception of freedom may be misleading in these patients. In KTRs who were aware of the presence of antibodies after vaccination, adherence decreased compared to those without an antibody response. We observed a dose-response effect, meaning rates of non-adherence are higher when the category of antibody response is higher. This is likely to be the result of the communication to participants that a higher antibody concentration is a correlate of protection. Moreover, differences in adherence were found between preventive measures. In comparison to non-responders, it seemed that low-responders were less adherent to preventive measures if they interfered with daily human-to-human interaction such as keeping distance and limiting visitors or visits, despite facing the risk of SARS-CoV-2 infection. Most importantly, higher adherence was associated with less risk of SARS-CoV-2 infection both before and after vaccination. Our data suggest that the adherence to preventive measures was protective against SARS-CoV-2 infection when vaccination was not yet available, but also in KTRs who received vaccination. Unfortunately, we were not able to assess whether the decreased adherence in antibody responders was justified due to too few SARS-CoV-2 infection events (N = 34) within the short timeframe (median 66 days) between awareness of antibody response and the event of a third vaccination.

Even though much attention has now been paid to the physical health consequences of COVID-19 in immunocompromised patient groups, mental health has unjustly received less attention. KTRs are not only likely to experience mental burden due to awareness of their susceptibility for a severe course of COVID-19 in case of infection, but also as a result of social isolation.^21^ In some cases, KTRs were isolated from household contacts or access to healthcare, and sometimes the same was true for their children as they were kept at home and received homeschooling for prolonged periods. Regarding employment in the Netherlands, vulnerable employees like immunocompromised patients are entitled, by law, to extra protection. This study provides argument advocating for but also against peak antibody measurement in KTRs^22^ and sharing this information with the patient. We believe that informing KTRs of their antibody response could improve a patients’ well-being in terms of mental health or quality of life. It could also be useful to identify and safely guide those still at risk of COVID-19 after vaccination. We see that although patients reach a certain antibody threshold and subsequently feel safe, antibody levels are still lower than in the general population,^10^ even after repeated vaccination.^23^, ^24^, ^25^, ^26^, ^27^ Therefore, patients are likely inadequately protected against severe COVID-19 if infection does occur, especially since there is no established threshold above which patients are deemed to be ‘safe’. Moreover, in practice it is likely that problems will be encountered, for instance the interpretation of results over time and dealing with heterogeneity between laboratories. The goal of measuring antibody levels in clinical practice is to give KTRs more freedom in terms of preventive measures as social interaction is essential to every aspect of our health. This however is challenging, since it is still unknown what antibody level is protective against COVID-19 in KTRs.

In this large-scale questionnaire study we are the first to show in KTRs, of all immunocompromised patient groups, the association between SARS-CoV-2 vaccination and awareness of subsequent antibody response, and the adherence to preventive measures. Furthermore, our data suggest that adherence to preventive measures effectively protects against SARS-CoV-2 infection. This was established in a patient group that tightly adhered to government recommendations overall. This tight adherence has previously been described in KTRs which resulted in no self-reported SARS-CoV-2 infections,^28^ and in other immunocompromised patients compared to the general population.^29^^,^^30^ A point of discussion is the communication of antibody response and its related protection against COVID-19. A patients’ ‘awareness of antibody response’ consists of their antibody level, corresponding category of antibody response, and a (very cautiously constructed) indication of protection against COVID-19. The latter in itself and not necessarily the awareness of antibody response could play a role in the registered behavioral changes. However, within this study design it is not possible to disentangle the solely effects of either antibody response or instructions. This study also has some limitations. At the time the present study was set up, no validated instrument to measure adherence to preventive measures was available. Therefore, we used self-designed questionnaires that were constructed together with the patient representatives of our consortium. We used repeated questionnaires to monitor the behavior of KTRs. However, we introduced recall bias when patients reported on adherence before SARS-CoV-2 vaccination at the time they already received their second vaccination, which was inherent to the study protocol as we included KTRs after they received two vaccinations. Subjective perceptions of infection or personal adherence to measures could also play a role. For instance, since SARS-CoV-2 infections were self-reported, it has the potential to confound results, but when patients are not aware of a possible infection, this is likely not to affect these associations. Furthermore, it can be argued that the relationship of adherence with infection rate is not solely based on adherence, as we were not able to correct for confounders due to too few infection events. Otherwise, potential confounders in the period before vaccination are: age, BMI, smoking behavior, eGFR and steroid use, and after vaccination: age, number of comorbidities and hypertension. However, at the same time, more external factors might have played a role in our analyses, but we can argue that these factors have not influenced the direction of our results. The first questionnaire on behavior was obtained from August till October 2021 during the Delta wave (government recommendations: slowly letting go of preventive measures), and the second questionnaire on behavior was obtained in December 2021 and January 2022, during the Omicron BA.1 wave (government recommendations: strict lockdown). It is uncertain how these external factors influenced the reported behavioral response in KTRs, as they were advised to very strictly adhere to (additional) preventive measures, and were sometimes even isolated from household contacts. We may argue that since each questionnaire period fell within one single wave, with similar governmental recommendations, these factors had a similar influence on reported behavioral responses. However, the second questionnaire was taken in a period of strict lockdown in which patients may have responded to adhere more tightly to restrictions compared to the first, independent of vaccination status and/or antibody response. Nonetheless, we found that patients adhered less tightly to restrictions when they were aware they had a higher antibody response, so the effect of awareness of antibody response on behavior is probably rather underestimated than overestimated during the Omicron BA.1 wave. Self-testing kits became available in the Netherlands in April 2021, however, we do not believe this to have affected self-reported infection rates. Participants of our cohort were already vigilant for possible infections, and were clearly instructed to perform a PCR test if they had respiratory symptoms. We also believe it is unlikely that the availability of self-testing kits has affected behavioral changes. KTRs were highly motivated to prevent a SARS-CoV-2 infection and the mere availability of these self-testing kits did not provide any safety or risk reduction. In addition, we do not expect the use of monoclonal antibodies to have affected behavioral changes in KTRs. Shortly after sotrovimab became available in the Netherlands, the SARS-CoV-2 Omicron variant was first detected, and quickly became the dominant variant. Since the less virulent course of this variant and reduced sensitivity to the neutralising effect of antibodies, sotrovimab was not considered necessary. The use of cilgavimab/tixagevimab was not utilised or recommended. Lastly, if we compare patients that were excluded for analysis (LESS CoV-2 exclusion cohorts as well as the national exclusion cohort) with the included patients, the latter were older, received more immunosuppressive agents and in comparison to the LESS CoV-2 exclusion cohorts, had more comorbidities. Because of this increased frailty we speculate that these included patients are likely to have adhered more tightly to preventive measures despite the development of an antibody response. Therefore, the observed association between antibody response and adherence might be an underestimation and not generalisable to all KTRs.

In conclusion, informing KTRs of the presence of antibodies after SARS-CoV-2 vaccination results in higher non-adherence to preventive measures and patients should be informed of the potential consequences. Moreover, because our findings insinuate that the adherence to government recommendations is effective in the prevention of SARS-CoV-2 infection. Future research is needed to find the optimal way to inform patients of individual vaccination results to improve quality of life without increasing the risk of infection due to inappropriate adherence to preventive measures.

## Contributors

The RECOVAC consortium and collaborators were involved in collection of the data. PB, ALM and MHH were mostly involved in collection of the LESS-CoV-2 study data. SCF, PB, ALM, JPMV, ACA, APJV, PTN, MHH, RTG, LBH, MEJR, JSFS, FJB and SEG were involved in the design of this study. SCF did the statistical analysis together with PB, PTN and ALM, who all accessed and verified the underlying data, and SCF drafted the article, whereas all authors took part in its revision. SCF had final responsibility for the decision to submit for publication and is the guarantor for the study.

## Data sharing statement

The data that support the findings of this study are available from the corresponding author, upon reasonable request. Research proposals can be submitted to the Consortium members via the corresponding author.

## Declaration of interests

We declare no competing interests.

## Appendix A Supplementary data

Supplemental file RECOVAC Collaborators

Supplementary Figures S1–S8 and Tables S1–S8

Translated_abstract
