# Supplementary material for: Adherence to preventive measures after SARS-CoV-2 vaccination and after awareness of antibody response in kidney transplant recipients in the Netherlands: a nationwide questionnaire study
Source: eClinicalMedicine. 2023 Jul 20;62:102103. doi: 10.1016/j.eclinm.2023.102103 (PMC10393559; doi:10.1016/j.eclinm.2023.102103)
Supplement: Supplemental file RECOVAC Collaborators [file mmc1.docx]

**RECOVAC Collaborators**

| **Forename initials** | **Surnames** |
| --- | --- |
| C. | Imhof |
| C. | Idzinga |
| C. | Siegert |
| C. C. | Baan |
| C. J. A. M. | Konings |
| C. | van Kessel |
| D. | van Baarle |
| D. A. | Diavatopoulos |
| D. | Standaar |
| E. | ten Hoope |
| E. | Til |
| E. B. M. | Remmerswaal |
| F. | van der Klis |
| H. R. | Fritsen |
| I. | Stijnman |
| J. N. | Brinkman |
| J. | Cheng |
| L. | den Biggelaar |
| M. | ten Dam |
| M. | Steenhuis |
| M. | Zwerink |
| M. H. J. | Braks |
| M. | Willems |
| M. L. | Kho |
| N. | Rots |
| P. | Vart |
| R. G. | van der Molen |
| R. M. A. | van den Dorpel |
| R. S. R. K. | Malaha |
| R. C. G. | ter Meulen |
| T. | Rispens |
| T. | Steenvoorden |
| T. | de Ronde |
| V. J. P. | Peters |
| W. S. | Konijn |
| W. M. T. | Janssen |
| W. J. | Bos |
| Y. M. R. | Adema |
| Y. | Vegting |
