# Supplementary material for: Adherence to preventive measures after SARS-CoV-2 vaccination and after awareness of antibody response in kidney transplant recipients in the Netherlands: a nationwide questionnaire study
Source: eClinicalMedicine. 2023 Jul 20;62:102103. doi: 10.1016/j.eclinm.2023.102103 (PMC10393559; doi:10.1016/j.eclinm.2023.102103)
Supplement: Supplementary Figures S1–S8 and Tables S1–S8 [file mmc2.pdf]

Table of contents

Table S1. Percentage of missingness for all descriptive variables and variables of interest

Table S2. Descriptive characteristics of in- and exclusion cohort after SARS-CoV-2 vaccination (analysis 1)

Table S3. Descriptive characteristics of in- and exclusion cohort after awareness of antibody response (analysis 2)

Table S4. Descriptive characteristics of the LESS CoV-2 inclusion cohort (analysis 1) and the national exclusion cohort

Table S5. Non-adherence to preventive measures after awareness of antibody response by group, corrected for confounders using three association models, including effect size of confounders

Table S6. Non-adherence to preventive measures after awareness of antibody response, taking the low-responder group as reference, corrected for confounders using three association models, including effect size of confounders

Table S7. Descriptive characteristics of KTRs according to SARS-CoV-2 infection before vaccination

Table S8. Descriptive characteristics of KTRs according to SARS-CoV-2 infection after vaccination

Figure S1. Overview of evolution COVID-19 pandemic during study period

Figure S2. Boxplots for average adherence scores for each of the preventive measures before and after SARS-CoV-2 vaccination in KTRs (N=2939)

Table S1. Percentage of missingness for all descriptive variables and variables of interest

	Missingness (%)
Male	0·0
European descent	4·1
Age, y	0·0
BMI, kg/m^2	3·5
Current smoking	2·3
Yes	
Past	
Never	
Current alcohol consumption	2·4
Daily	
Less than daily	
Never	
Primary renal diagnosis	18·7
Congenital/hereditary disease	
Cystic kidney disease	
Diabetic kidney disease	
Glomerulonephritis	
Interstitial nephritis/ pyelonephritis/drug induced nefropathy/urolithiasis	
Renal vascular disease	
Other multisystemic disease	
Other	
Unknown	
No. of comorbidities	0·0
None	
1	
2	
≥3	
Comorbidities	0·0
Diabetes mellitus	
Chronic lung disease	
Heart failure	
Hypertension	
Coronary artery disease	
Peripheral vascular disease	
Malignancy	
Stroke	
Dementia	
Liver cirrhosis	
HIV/AIDS	
eGFR, mL/min/1·73m^2	20·9
Transplant characteristics	20·9
Transplantation type	
DBD	
DCD	
Living donation	
First kidney transplant	
Time after transplantation, y	
Graft failure	
Vaccine type	2·3
mRNA-1273	
BNT162b2	
ChAdOx-nCov19	
Ad26.CoV2.S	
No. of immunosuppressive agents	0·0
None	
1	
2	
≥3	
Immunosuppressive treatment	0·0
Steroids	
Calcineurin inhibitor	
MMF/MPA	
Azathioprine	
mTOR inhibitor	
Antibody level, BAU/mL	0·0
Adherence	
Before vaccination	3·5

**After vaccination**	5·9
**After antibody awareness**	4·7
**Previous SARS-CoV-2 infection**	
**Before vaccination**	2·4
**After vaccination**	2·4

Abbreviations are: BMI, body mass index; HIV/AIDS, human immunodeficiency virus/acquired immunodeficiency syndrome; eGFR, estimated glomerular filtration rate; DBD, donation after brain death; DCD, donation after circulatory death; MMF/MPA, mycophenolate mofetil/mycophenolic acid; mTOR, mammalian target of rapamycin; BAU, binding antibody units; SARS-CoV-2, severe acute respiratory syndrome coronavirus-2

Table S2. Descriptive characteristics of in- and exclusion cohort after SARS-CoV-2 vaccination (analysis 1)

	Inclusion cohort (N=2939)	Exclusion cohort (N=592)	p-value[a]
**Male, n (%)**	1704 (58·0)	386 (65·2)	0·001
**European descent, n (%)**	2595 (91·9)	308 (84·6)	<0·001
**Age, y**	58·7 (12·4)	54·4 (17·4)	<0·001
**BMI, kg/m^2**	26·0 (4·5)	26·3 (4·9)	0·286
**Current smoking, n (%)**			0·255
**Yes**	167 (5·8)	30 (7·9)	
**Past**	1342 (46·7)	175 (46·3)	
**Never**	1367 (47·5)	173 (45·8)	
**Current alcohol consumption, n (%)**			0·780
**Daily**	1633 (56·9)	220 (58·0)	
**Less than daily**	1006 (35·0)	132 (34·8)	
**Never**	233 (8·1)	27 (7·1)	
**Primary renal diagnosis, n (%)**			0·523
**Congenital/hereditary disease**	80 (3·6)	12 (2·7)	
**Cystic kidney disease**	393 (17·6)	60 (13·7)	
**Diabetic kidney disease**	148 (6·6)	33 (7·5)	
**Glomerulonephritis**	459 (20·5)	91 (20·8)	
**Interstitial nephritis/ pyelonephritis/drug induced nefropathy/urolithiasis**	163 (7·3)	27 (6·2)	
**Renal vascular disease**	159 (7·1)	36 (8·2)	
**Other multisystemic disease**	114 (5·1)	24 (5·5)	
**Other**	595 (26·6)	130 (29·7)	
**Unknown**	124 (5·5)	25 (5·7)	
**No. of comorbidities, n (%)**			<0·001
**None**	380 (12·9)	246 (41·7)	
**1**	1421 (48·3)	177 (30·0)	
**2**	729 (24·8)	110 (18·6)	
**≥3**	409 (13·9)	57 (9·7)	
**Comorbidities, n (%)**			
**Diabetes mellitus**	738 (25·1)	117 (19·8)	0·007
**Chronic lung disease**	196 (6·7)	27 (4·6)	0·070
**Heart failure**	181 (6·2)	22 (3·7)	0·027
**Hypertension**	2423 (82·4)	317 (53·7)	<0·001
**Coronary artery disease**	345 (11·7)	47 (8·0)	0·010
**Peripheral vascular disease**	112 (3·8)	13 (2·2)	0·071
**Malignancy**	92 (3·1)	11 (1·9)	0·125
**Stroke**	144 (4·9)	26 (4·4)	0·686
**Dementia**	1 (0·0)	1 (0·2)	0·754
**Liver cirrhosis**	24 (0·8)	5 (0·8)	1·000
**HIV/AIDS**	6 (0·2)	0 (0·0)	0·582
**eGFR, mL/min/1·73m^2**	50·7 (19·0)	52·0 (18·5)	0·180
**Transplant characteristics**			
**Transplantation type**			0·039
**DBD**	491 (22·6)	78 (18·9)	
**DCD**	307 (14·1)	76 (18·4)	
**Living donation**	1377 (63·3)	259 (62·7)	
**First kidney transplant, n (%)**	1877 (86·3)	344 (83·3)	0·126
**Time after transplantation, y**	7·9 [4·1, 13·9]	6·8 [3·6, 12·3]	0·005
**Graft failure, n (%)**	182 (8·4)	25 (6·1)	0·136
**Vaccine type, n (%)**			0·107
**mRNA-1273**	2647 (92·0)	336 (88·7)	
**BNT162b2**	158 (5·5)	30 (7·9)	
**ChAdOx-nCov19**	69 (2·4)	12 (3·2)	
**Ad26.CoV2.S**	1 (0·0)	0 (0·0)	
**Unknown**	1 (0·0)	1 (0·3)	
**No. of immunosuppressive agents, n (%)**			<0·001
**None**	114 (3·9)	218 (36·9)	
**1**	295 (10·0)	51 (8·6)	
**2**	1505 (51·2)	186 (31·5)	
**≥3**	1025 (34·9)	135 (22·9)	
**Immunosuppressive treatment, n (%)**			
**Steroids**	1972 (67·1)	237 (40·2)	<0·001
**Calcineurin inhibitor**	2299 (78·2)	305 (51·7)	<0·001
**MMF/MPA**	1657 (56·4)	231 (39·2)	<0·001
**Azathioprine**	292 (9·9)	35 (5·9)	0·003
**mTOR inhibitor**	177 (6·0)	26 (4·4)	0·150

[a] p-values were calculated using independent sample t test for normally distributed continuous variables, Mann-Whitney U test in case of non-normally distributed continuous variables and chi-square test in case of categorical variables.

Due to missing values total numbers and values can vary. No missings for sex and age.

Abbreviations are: SARS-CoV-2, severe acute respiratory syndrome coronavirus-2; BMI, body mass index; HIV/AIDS, human immunodeficiency virus/acquired immunodeficiency syndrome; eGFR, estimated glomerular filtration rate; DBD, donation after brain death; DCD, donation after circulatory death; MMF/MPA, mycophenolate mofetil/mycophenolic acid; mTOR, mammalian target of rapamycin

Table S3. Descriptive characteristics of in- and exclusion cohort after awareness of antibody response (analysis 2)

	Inclusion cohort (N=2399)	Exclusion cohort (N=1132)	p-value[a]
**Male, n (%)**	1369 (57·1)	721 (63·7)	<0·001
**European descent, n (%)**	2139 (92·4)	764 (87·7)	<0·001
**Age, y**	59·2 (12·2)	55·4 (15·4)	<0·001
**BMI, kg/m^2**	26·0 (4·3)	26·1 (5·0)	0·566
**Current smoking, n (%)**			0·138
**Yes**	136 (5·8)	61 (6·8)	
**Past**	1125 (47·6)	392 (44·0)	
**Never**	1102 (46·6)	438 (49·2)	
**Current alcohol consumption, n (%)**			0·062
**Daily**	1326 (56·2)	527 (59·1)	
**Less than daily**	830 (35·2)	308 (34·6)	
**Never**	204 (8·6)	56 (6·3)	
**Primary renal diagnosis, n (%)**			0·259
**Congenital/hereditary disease**	73 (3·7)	19 (2·6)	
**Cystic kidney disease**	348 (17·9)	105 (14·5)	
**Diabetic kidney disease**	127 (6·5)	54 (7·5)	
**Glomerulonephritis**	386 (19·8)	164 (22·7)	
**Interstitial nephritis/ pyelonephritis/drug induced nefropathy/urolithiasis**	141 (7·2)	49 (6·8)	
**Renal vascular disease**	146 (7·5)	49 (6·8)	
**Other multisystemic disease**	103 (5·3)	35 (4·8)	
**Other**	521 (26·7)	204 (28·2)	
**Unknown**	104 (5·3)	45 (6·2)	
**No. of comorbidities, n (%)**			<0·001
**None**	290 (12·1)	336 (29·7)	
**1**	1171 (48·8)	427 (37·8)	
**2**	606 (25·3)	233 (20·6)	
**≥3**	332 (13·8)	134 (11·9)	
**Comorbidities, n (%)**			
**Diabetes mellitus**	592 (24·7)	263 (23·3)	0·387
**Chronic lung disease**	170 (7·1)	53 (4·7)	0·008
**Heart failure**	147 (6·1)	56 (5·0)	0·188
**Hypertension**	1998 (83·3)	742 (65·7)	<0·001
**Coronary artery disease**	281 (11·7)	111 (9·8)	0·107
**Peripheral vascular disease**	88 (3·7)	37 (3·3)	0·622
**Malignancy**	77 (3·2)	26 (2·3)	0·165
**Stroke**	126 (5·3)	44 (3·9)	0·094
**Dementia**	1 (0·0)	1 (0·1)	1·000
**Liver cirrhosis**	20 (0·8)	9 (0·8)	1·000
**HIV/AIDS**	6 (0·3)	0 (0·0)	0·213
**eGFR, mL/min/1·73m^2**	50·8 (18·8)	51·1 (19·0)	0·668
**Transplant characteristics**			
**Transplantation type**			0·391
**DBD**	429 (22·7)	140 (20·2)	
**DCD**	276 (14·6)	107 (15·4)	
**Living donation**	1189 (62·8)	447 (64·4)	
**First kidney transplant, n (%)**	1635 (86·3)	586 (84·4)	0·248
**Time after transplantation, y**	7·9 [4·1, 13·9]	7·3 [3·7, 13·0]	0·059
**Graft failure, n (%)**	163 (8·6)	44 (6·3)	0·072
**Vaccine type, n (%)**			0·052
**mRNA-1273**	2155 (91·2)	828 (92·7)	
**BNT162b2**	140 (5·9)	48 (5·4)	
**ChAdOx-nCov19**	66 (2·8)	15 (1·7)	
**Ad26.CoV2.S**	1 (0·0)	0 (0·0)	
**Unknown**	0 (0·0)	2 (0·2)	
**No. of immunosuppressive agents, n (%)**			<0·001
**None**	82 (3·4)	250 (22·1)	
**1**	265 (11·0)	81 (7·2)	
**2**	1222 (50·9)	469 (41·5)	
**≥3**	830 (34·6)	330 (29·2)	
**Immunosuppressive treatment, n (%)**			
**Steroids**	1615 (67·3)	594 (52·6)	<0·001
**Calcineurin inhibitor**	1872 (78·0)	732 (64·8)	<0·001
**MMF/MPA**	1322 (55·1)	566 (50·1)	0·006
**Azathioprine**	244 (10·2)	83 (7·3)	0·008
**mTOR inhibitor**	160 (6·7)	43 (3·8)	0·001

[a] p-values were calculated using independent sample t test for normally distributed continuous variables, Mann-Whitney U test in case of non-normally distributed continuous variables and chi-square test in case of categorical variables.

Due to missing values total numbers and values can vary. No missings for sex and age.

Abbreviations are: BMI, body mass index; HIV/AIDS, human immunodeficiency virus/acquired immunodeficiency syndrome; eGFR, estimated glomerular filtration rate; DBD, donation after brain death; DCD, donation after circulatory death; MMF/MPA, mycophenolate mofetil/mycophenolic acid; mTOR, mammalian target of rapamycin

Table S4. Descriptive characteristics of the LESS CoV-2 inclusion cohort (analysis 1) and the national exclusion cohort

	LESS CoV-2 inclusion cohort (N=2939)	National exclusion cohort (N=12·471)[a]	p-value[b]
**Male, n (%)**	1704 (58·0)	7469 (59·9)	0·060
**Age, y**	50·7 (12·4)*	47·4 (16·1)	<0·001
**Primary renal diagnosis, n (%)**			<0·001
**Congenital/hereditary disease**	80 (3·6)	520 (5·5)	
**Cystic kidney disease**	393 (17·6)	1412 (14·8)	
**Diabetic kidney disease**	148 (6·6)	787 (8·3)	
**Glomerulonephritis**	459 (20·5)	1536 (16·1)	
**Interstitial nephritis/ pyelonephritis/drug induced nefropathy/urolithiasis**	163 (7·3)	840 (8·8)	
**Renal vascular disease**	159 (7·1)	1254 (13·2)	
**Other multisystemic disease**	114 (5·1)	615 (6·5)	
**Other**	595 (26·6)	1108 (11·6)	
**Unknown**	124 (5·5)	1457 (15·3)	
**Creatinine, µmol/L**	136·7 (70·0)	130·3 (76·7)*	0·001
**Transplant characteristics**			
**Transplantation type**			<0·001
**DBD**	491 (22·6)	2975 (23·9)	
**DCD**	307 (14·1)	2716 (21·8)	
**Living donation**	1377 (63·3)	6780 (54·4)	
**First kidney transplant, n (%)**	1877 (86·3)	10668 (85·5)	0·371
**Time after transplantation, y**	7·9 [4·1, 13·9]	*	*
**Graft failure, n (%)**	182 (8·4)	2219 (17·8)	<0·001
**No. of immunosuppressive agents, n (%)**		*	<0·001
**None**	114 (3·9)	179 (3·5)	
**1**	295 (10·0)	3128 (61·7)	
**2**	1505 (51·2)	1726 (34·0)	
**≥3**	1025 (34·9)	40 (0·8)	
**Immunosuppressive treatment, n (%)**		*	
**Steroids**	1972 (67·1)	3723 (73·4)	<0·001
**Calcineurin inhibitor**	2299 (78·2)	3823 (75·4)	0·004
**MMF/MPA**	1657 (56·4)	3222 (63·5)	<0·001
**Azathioprine**	292 (9·9)	463 (9·1)	0·248
**mTOR inhibitor**	177 (6·0)	228 (4·5)	0·003

Of note: Descriptive characteristics of the national exclusion cohort are derived from the Dutch Organ Transplantation Registry (NOTR), which registers demographic data of ~95% of the total kidney transplant population in the Netherlands since the year 2000, included at the moment of transplantation and with an annual follow-up. We extracted all available data from kidney transplant recipients (KTRs) that were alive up to and including the end of our study period (2021). The LESS CoV-2 inclusion cohort is part of the national exclusion cohort.

* All characteristics, except for the use of immunosuppressive agents and creatinine level, were obtained at the moment of transplantation. Since we present our inclusion cohort at a median follow-up time of 8 years after transplantation, we selected these characteristics at a follow-up of 8 years in the NOTR data as well. At 8 years follow-up, this data is available in 5073 participants. Consistently, we also subtracted 8 years from the mean age of our inclusion cohort.

[a] A larger number of KTRs was analysed than we invited to participate, as we compare our inclusion cohort with patients from the NOTR who were 8 years after transplantation during the period 2000-2021.

[b] p-values were calculated using independent sample t test for normally distributed continuous variables, Mann-Whitney U test in case of non-normally distributed continuous variables and chi-square test in case of categorical variables.

Due to missing values total numbers and values can vary. No missings in inclusion cohort for gender and age.

Abbreviations are: DBD, donation after brain death; DCD, donation after circulatory death; MMF/MPA, mycophenolate mofetil/mycophenolic acid; mTOR, mammalian target of rapamycin

Table S5. Non-adherence to preventive measures after awareness of antibody response by group, corrected for confounders using three association models, including effect size of confounders

	Model 0[a]		Model 1		Model 2	
	**OR [95% CI]**	**p-value[b]**	**OR [95% CI]**	**p-value**	**OR [95% CI]**	**p-value**
**Keep 1.5m distance**						
**Antibody group**						
- Non-responder	ref.	ref.	ref.	ref.	ref.	ref.
- Low-responder	1·49 [1·21-1·83]	<·001	1·43 [1·16-1·76]	<·001	1·42 [1·16-1·75]	<·001
- High-responder	2·61 [2·19-3·12]	<·001	2·36 [1·98-2·82]	<·001	2·12 [1·77-2·56]	<·001
**Age**			0·98 [0·97-0·98]	<·001	0·98 [0·97-0·98]	<·001
**Previous infection**					1·94 [1·45-2·59]	<·001
**Wear a face mask**						
**Antibody group**						
- Non-responder	ref.	ref.	ref.	ref.	ref.	ref.
- Low-responder	1·30 [1·01-1·68]	0·042	1·26 [0·97-1·62]	0·078	1·25 [0·97-1·62]	0·086
- High-responder	1·64 [1·33-2·02]	<·001	1·47 [1·19-1·82]	<·001	1·39 [1·12-1·74]	0·003
**Age**			0·98 [0·97-0·98]	<·001	0·98 [0·97-0·98]	<·001
**Previous infection**					1·21 [0·87-1·67]	0·243
**Hand washing**						
**Antibody group**						
- Non-responder	ref.	ref.	ref.	ref.	ref.	ref.
- Low-responder	1·05 [0·85-1·29]	0·642	1·03 [0·84-1·26]	0·801	1·05 [0·85-1·29]	0·639
- High-responder	1·64 [1·38-1·95]	<·001	1·54 [1·30-1·84]	<·001	1·45 [1·21-1·74]	<·001
**Age**			0·99 [0·98-0·99]	<·001	0·99 [0·98-0·99]	<·001
**Previous infection**					1·49 [1·12-1·97]	0·006
**Avoid supermarket or shops**						
**Antibody group**						
- Non-responder	ref.	ref.	ref.	ref.	ref.	ref.
- Low-responder	1·44 [1·18-1·75]	<·001	1·37 [1·13-1·67]	0·001	1·39 [1·14-1·69]	0·001
- High-responder	2·37 [2·02-2·79]	<·001	2·09 [1·77-2·47]	<·001	1·98 [1·67-2·35]	<·001
**Age**			0·97 [0·96-0·98]	<·001	0·97 [0·96-0·98]	<·001
**Previous infection**					1·34 [1·03-1·76]	0·032
**Avoid public transport**						
**Antibody group**						
- Non-responder	ref.	ref.	ref.	ref.	ref.	ref.
- Low-responder	1·29 [0·98-1·69]	0·069	1·24 [0·94-1·63]	0·126	1·24 [0·94-1·63]	0·132
- High-responder	1·81 [1·45-2·26]	<·001	1·58 [1·26-1·98]	<·001	1·51 [1·19-1·91]	<·001
**Age**			0·97 [0·97-0·98]	<·001	0·97 [0·97-0·98]	<·001
**Previous infection**					1·24 [0·84-1·80]	0·269
**Avoid crowded places**						
**Antibody group**						
- Non-responder	ref.	ref.	ref.	ref.	ref.	ref.
- Low-responder	1·43 [1·11-1·84]	0·006	1·32 [1·02-1·71]	0·035	1·31 [1·01-1·71]	0·041
- High-responder	2·76 [2·25-3·39]	<·001	2·28 [1·85-2·82]	<·001	2·07 [1·66-2·58]	<·001
**Age**			0·95 [0·95-0·96]	<·001	0·95 [0·95-0·96]	<·001
**Previous infection**					1·59 [1·17-2·16]	0·003
**Limit visitors or visits**						
**Antibody group**						

- Non-responder	ref.	ref.	ref.	ref.	ref.	ref.
- Low-responder	1·25 [1·03-1·52]	0·025	1·23 [1·01-1·50]	0·039	1·23 [1·01-1·50]	0·042
- High-responder	1·46 [1·24-1·72]	<·001	1·37 [1·16-1·62]	<·001	1·30 [1·10-1·55]	0·003
**Age**			0·99 [0·98-0·99]	<·001	0·99 [0·98-0·99]	<·001
**Previous infection**					1·40 [1·06-1·85]	0·017
**Work from home**						
**Antibody group**						
- Non-responder	ref.	ref.	ref.	ref.	ref.	ref.
- Low-responder	1·13 [0·82-1·55]	0·465	1·11 [0·81-1·53]	0·523	1·11 [0·80-1·53]	0·520
- High-responder	1·62 [1·26-2·10]	<·001	1·51 [1·17-1·97]	0·002	1·34 [1·02-1·76]	0·035
**Age**			0·99 [0·98-0·99]	0·003	0·98 [0·97-0·99]	<·001
**Previous infection**					1·95 [1·32-2·89]	<·001
**Avoid travel abroad**						
**Antibody group**						
- Non-responder	ref.	ref.	ref.	ref.	ref.	ref.
- Low-responder	1·19 [0·90-1·57]	0·231	1·15 [0·86-1·52]	0·338	1·14 [0·85-1·51]	0·373
- High-responder	1·76 [1·40-2·20]	<·001	1·56 [1·24-1·97]	<·001	1·52 [1·19-1·93]	<·001
**Age**			0·98 [0·97-0·98]	<·001	0·98 [0·97-0·98]	<·001
**Previous infection**					1·15 [0·79-1·65]	0·452

[a] Model 0: crude

Model 1: age

Model 2: age, previous severe acute respiratory syndrome coronavirus-2 infection

[b] p-values were calculated using ordinal logistic regression analysis.

Abbreviations are: OR, odds ratio; CI, confidence interval

Table S6. Non-adherence to preventive measures after awareness of antibody response, taking the low-responder group as reference, corrected for confounders using three association models, including effect size of confounders

	Model 0[a]		Model 1		Model 2	
	OR [95% CI]	p-value[b]	OR [95% CI]	p-value	OR [95% CI]	p-value
**Keep 1.5m distance**						
**Antibody group**						
- Low-responder	ref.	ref.	ref.	ref.	ref.	ref.
- High-responder	1·75 [1·42-2·15]	<·001	1·65 [1·34-2·03]	<·001	1·50 [1·21-1·86]	<·001
**Age**			0·98 [0·97-0·98]	<·001	0·98 [0·97-0·99]	<·001
**Previous infection**					1·84 [1·36-2·50]	<·001
**Wear a face mask**						
**Antibody group**						
- Low-responder	ref.	ref.	ref.	ref.	ref.	ref.
- High-responder	1·25 [0·98-1·60]	0·068	1·17 [0·91-1·50]	0·222	1·12 [0·87-1·45]	0·376
**Age**			0·98 [0·97-0·99]	<·001	0·98 [0·97-0·99]	<·001
**Previous infection**					1·13 [0·80-1·59]	0·481
**Hand washing**						
**Antibody group**						
- Low-responder	ref.	ref.	ref.	ref.	ref.	ref.
- High-responder	1·54 [1·26-1·89]	<·001	1·49 [1·21-1·83]	<·001	1·38 [1·12-1·71]	0·003
**Age**			0·99 [0·98-0·99]	0·002	0·99 [0·98-0·99]	0·008
**Previous infection**					1·41 [1·05-1·90]	0·023
**Avoid supermarket or shops**						
**Antibody group**						
- Low-responder	ref.	ref.	ref.	ref.	ref.	ref.
- High-responder	1·65 [1·36-2·00]	<·001	1·54 [1·27-1·87]	<·001	1·46 [1·19-1·79]	<·001
**Age**			0·97 [0·97-0·98]	<·001	0·97 [0·97-0·98]	<·001
**Previous infection**					1·22 [0·92-1·63]	0·161
**Avoid public transport**						
**Antibody group**						
- Low-responder	ref.	ref.	ref.	ref.	ref.	ref.
- High-responder	1·40 [1·07-1·83]	0·014	1·26 [0·96-1·66]	0·094	1·21 [0·92-1·61]	0·180
**Age**			0·97 [0·96-0·98]	<·001	0·97 [0·96-0·98]	<·001
**Previous infection**					1·22 [0·82-1·81]	0·322
**Avoid crowded places**						
**Antibody group**						
- Low-responder	ref.	ref.	ref.	ref.	ref.	ref.
- High-responder	1·91 [1·51-2·43]	<·001	1·71 [1·35-2·19]	<·001	1·56 [1·21-2·01]	<·001
**Age**			0·95 [0·95-0·96]	<·001	0·95 [0·94-0·96]	<·001
**Previous infection**					1·62 [1·18-2·22]	0·003
**Limit visitors or visits**						
**Antibody group**						
- Low-responder	ref.	ref.	ref.	ref.	ref.	ref.
- High-responder	1·17 [0·96-1·42]	0·120	1·12 [0·92-1·36]	0·266	1·07 [0·87-1·31]	0·533
**Age**			0·99 [0·98-0·99]	<·001	0·99 [0·98-0·99]	<·001
**Previous infection**					1·40 [1·05-1·88]	0·021
**Work from home**						

**Antibody group**						
- Low-responder	ref.	ref.	ref.	ref.	ref.	ref.
- High-responder	1·46 [1·08-1·98]	0·015	1·39 [1·02-1·89]	0·037	1·21 [0·88-1·67]	0·238
**Age**			0·99 [0·98-0·99]	0·028	0·98 [0·97-0·99]	0·009
**Previous infection**					2·03 [1·34-3·08]	<·001
**Avoid travel abroad**						
**Antibody group**						
- Low-responder	ref.	ref.	ref.	ref.	ref.	ref.
- High-responder	1·48 [1·13-1·94]	0·004	1·37 [1·05-1·80]	0·023	1·35 [1·02-1·80]	0·035
**Age**			0·98 [0·97-0·99]	<·001	0·98 [0·97-0·99]	<·001
**Previous infection**					1·10 [0·75-1·61]	0·617

[a] Model 0: crude

Model 1: age

Model 2: age, previous severe acute respiratory syndrome coronavirus-2 infection

[b] p-values were calculated using ordinal logistic regression analysis.

Abbreviations are: OR, odds ratio; CI, confidence interval

Table S7. Descriptive characteristics of KTRs according to SARS-CoV-2 infection before vaccination

	SARS-CoV-2 infection (N=202)	No SARS-CoV-2 infection (N=2666)	p-value[b]
**Male, n (%)**	110 (54·5)	1549 (58·1)	0·348
**European descent, n (%)[a]**	180 (90·5)	2407 (92·0)	0·521
**Age, y**	56·9 (11·6)	58·8 (12·4)	0·039
**BMI, kg/m^{2}[a]**	27·0 (4·8)	26·0 (4·4)	0·002
**Current smoking, n (%)[a]**			0·010
**Yes**	2 (1·0)	163 (6·1)	
**Past**	97 (48·0)	1241 (46·6)	
**Never**	103 (51·0)	1260 (47·3)	
**Current alcohol consumption, n (%)[a]**			0·139
**Daily**	125 (61·9)	1500 (56·4)	
**Less than daily**	67 (33·2)	937 (35·2)	
**Never**	10 (5·0)	223 (8·4)	
**Primary renal diagnosis, n (%)[a]**			0·225
**Congenital/hereditary disease**	10 (6·0)	68 (3·4)	
**Cystic kidney disease**	33 (19·9)	349 (17·4)	
**Diabetic kidney disease**	10 (6·0)	132 (6·6)	
**Glomerulonephritis**	35 (21·1)	409 (20·4)	
**Interstitial nephritis/ pyelonephritis/drug induced nefropathy/urolithiasis**	12 (7·2)	148 (7·4)	
**Renal vascular disease**	4 (2·4)	153 (7·6)	
**Other multisystemic disease**	6 (3·6)	106 (5·3)	
**Other**	45 (27·1)	531 (26·5)	
**Unknown**	11 (6·6)	110 (5·5)	
**No. of comorbidities, n (%)**			0·629
**None**	27 (13·4)	291 (10·9)	
**1**	93 (46·0)	1324 (49·7)	
**2**	51 (25·2)	676 (25·4)	
**≥3**	31 (15·3)	375 (14·1)	
**Comorbidities, n (%)**			
**Diabetes mellitus**	62 (30·7)	673 (25·2)	0·104
**Chronic lung disease**	16 (7·9)	178 (6·7)	0·594
**Heart failure**	13 (6·4)	167 (6·3)	1·000
**Hypertension**	163 (80·7)	2251 (84·4)	0·192
**Coronary artery disease**	23 (11·4)	322 (12·1)	0·858
**Peripheral vascular disease**	13 (6·4)	96 (3·6)	0·066
**Malignancy**	8 (4·0)	83 (3·1)	0·650
**Stroke**	6 (3·0)	138 (5·2)	0·224
**Dementia**	0 (0·0)	1 (0·0)	1·000
**Liver cirrhosis**	0 (0·0)	24 (0·9)	0·340
**HIV/AIDS**	0 (0·0)	6 (0·2)	1·000
**eGFR, mL/min/1·73m^{2}[a]**	46·8 (16·8)	50·9 (19·1)	0·009
**Transplant characteristics[a]**			
**Transplantation type**			0·426
**DBD**	30 (18·5)	444 (22·7)	
**DCD**	22 (13·6)	274 (14·0)	
**Living donation**	110 (67·9)	1236 (63·3)	
**First kidney transplant, n (%)**	140 (86·4)	1685 (86·2)	1·000
**Time after transplantation, y**	8·0 [3·7, 14·8]	8·0 [4·2, 13·9]	0·559
**Graft failure, n (%)**	14 (8·6)	164 (8·4)	1·000
**Vaccine type, n (%)[a]**			0·383
**mRNA-1273**	181 (89·6)	2459 (92·2)	
**BNT162b2**	12 (5·9)	145 (5·4)	
**ChAdOx-nCov19**	9 (4·5)	60 (2·3)	
**Ad26.CoV2.S**	0 (0·0)	1 (0·0)	
**No. of immunosuppressive agents, n (%)**			0·420
**None**	3 (1·5)	49 (1·8)	
**1**	14 (6·9)	280 (10·5)	
**2**	111 (55·0)	1388 (52·1)	
**≥3**	74 (36·6)	949 (35·6)	
**Immunosuppressive treatment, n (%)**			
**Steroids**	152 (75·2)	1813 (68·0)	0·040
**Calcineurin inhibitor**	163 (80·7)	2128 (79·8)	0·836
**MMF/MPA**	108 (53·5)	1545 (58·0)	0·242
**Azathioprine**	23 (11·4)	269 (10·1)	0·641
**mTOR inhibitor**	17 (8·4)	160 (6·0)	0·221
**Antibody level, BAU/mL**	3526·5 [1647·9, 7916·6]	92·8 [10·3, 773·7]	<0·001

[a] Due to missing values total numbers and values can vary.
[b] p-values were calculated using independent sample t test for normally distributed continuous variables, Mann-Whitney U test in case of non-normally distributed continuous variables and chi-square test in case of categorical variables.

Abbreviations are: KTRs, kidney transplant recipients; SARS-CoV-2, severe acute respiratory syndrome coronavirus-2; BMI, body mass index; HIV/AIDS, human immunodeficiency virus/acquired immunodeficiency syndrome; eGFR, estimated glomerular filtration rate; DBD, donation after brain death; DCD, donation after circulatory death; MMF/MPA, mycophenolate mofetil/mycophenolic acid; mTOR, mammalian target of rapamycin; BAU, binding antibody units

Table S8. Descriptive characteristics of KTRs according to SARS-CoV-2 infection after vaccination

	SARS-CoV-2 infection (N=27)	No SARS-CoV-2 infection (N=2841)	p-value[b]
**Male, n (%)**	12 (44·4)	1647 (58·0)	0·222
**European descent, n (%)[a]**	24 (100·0)	2561 (91·8)	0·278
**Age, y**	52·0 (14·6)	58·7 (12·3)	0·005
**BMI, kg/m^{2}[a]**	26·9 (4·5)	26·0 (4·5)	0·331
**Current smoking, n (%)[a]**			0·366
**Yes**	3 (11·5)	163 (5·7)	
**Past**	13 (50·0)	1324 (46·6)	
**Never**	10 (38·5)	1352 (47·6)	
**Current alcohol consumption, n (%)[a]**			0·282
**Daily**	15 (57·7)	1610 (56·8)	
**Less than daily**	11 (42·3)	992 (35·0)	
**Never**	0 (0·0)	233 (8·2)	
**Primary renal diagnosis, n (%)[a]**			0·438
**Congenital/hereditary disease**	0 (0·0)	78 (3·6)	
**Cystic kidney disease**	5 (22·7)	377 (17·5)	
**Diabetic kidney disease**	4 (18·2)	137 (6·4)	
**Glomerulonephritis**	3 (13·6)	442 (20·5)	
**Interstitial nephritis/ pyelonephritis/drug induced nefropathy/urolithiasis**	1 (4·5)	159 (7·4)	
**Renal vascular disease**	2 (9·1)	156 (7·3)	
**Other multisystemic disease**	2 (9·1)	110 (5·1)	
**Other**	4 (18·2)	572 (26·6)	
**Unknown**	1 (4·5)	120 (5·6)	
**No. of comorbidities, n (%)**			0·002
**None**	9 (33·3)	309 (10·9)	
**1**	7 (25·9)	1408 (49·6)	
**2**	7 (25·9)	721 (25·4)	
**≥3**	4 (14·8)	403 (14·2)	
**Comorbidities, n (%)**			
**Diabetes mellitus**	10 (37·0)	727 (25·6)	0·257
**Chronic lung disease**	1 (3·7)	194 (6·8)	0·796
**Heart failure**	2 (7·4)	178 (6·3)	1·000
**Hypertension**	16 (59·3)	2398 (84·4)	0·001
**Coronary artery disease**	3 (11·1)	341 (12·0)	1·000
**Peripheral vascular disease**	1 (3·7)	109 (3·8)	1·000
**Malignancy**	0 (0·0)	92 (3·2)	0·688
**Stroke**	1 (3·7)	143 (5·0)	1·000
**Dementia**	0 (0·0)	1 (0·0)	1·000
**Liver cirrhosis**	1 (3·7)	23 (0·8)	0·561
**HIV/AIDS**	0 (0·0)	6 (0·2)	1·000
**eGFR, mL/min/1·73m^{2}[a]**	54·6 (22·0)	50·6 (19·0)	0·386
**Transplant characteristics[a]**			
**Transplantation type**			0·657
**DBD**	5 (29·4)	470 (22·4)	
**DCD**	3 (17·6)	293 (14·0)	
**Living donation**	9 (52·9)	1337 (63·7)	
**First kidney transplant, n (%)**	16 (94·1)	1810 (86·2)	0·554
**Time after transplantation, y**	4·6 [3·2, 11·1]	8·0 [4·1, 13·9]	0·303
**Graft failure, n (%)**	2 (11·8)	175 (8·3)	0·945
**Vaccine type, n (%)[a]**			0·972
**mRNA-1273**	23 (88·5)	2615 (92·1)	
**BNT162b2**	2 (7·7)	155 (5·5)	
**ChAdOx-nCov19**	1 (3·8)	68 (2·4)	
**Ad26.CoV2.S**	0 (0·0)	1 (0·0)	
**No. of immunosuppressive agents, n (%)**			0·161
**None**	2 (7·4)	52 (1·8)	
**1**	2 (7·4)	291 (10·2)	
**2**	12 (44·4)	1488 (52·4)	
**≥3**	11 (40·7)	1010 (35·6)	
**Immunosuppressive treatment, n (%)**			
**Steroids**	18 (66·7)	1946 (68·5)	1·000
**Calcineurin inhibitor**	21 (77·8)	2269 (79·9)	0·977
**MMF/MPA**	16 (59·3)	1635 (57·6)	1·000
**Azathioprine**	5 (18·5)	286 (10·1)	0·260
**mTOR inhibitor**	1 (3·7)	176 (6·2)	0·894
**Antibody level, BAU/mL**	232·7 [10·0, 2373·5]	122·2 [12·2, 1124·5]	0·552

[a] Due to missing values total numbers and values can vary.
[b] p-values were calculated using independent sample t test for normally distributed continuous variables, Mann-Whitney U test in case of non-normally distributed continuous variables and chi-square test in case of categorical variables.

Abbreviations are: KTRs, kidney transplant recipients; SARS-CoV-2, severe acute respiratory syndrome coronavirus-2; BMI, body mass index; HIV/AIDS, human immunodeficiency virus/acquired immunodeficiency syndrome; eGFR, estimated glomerular filtration rate; DBD, donation after brain death; DCD, donation after circulatory death; MMF/MPA, mycophenolate mofetil/mycophenolic acid; mTOR, mammalian target of rapamycin; BAU, binding antibody units

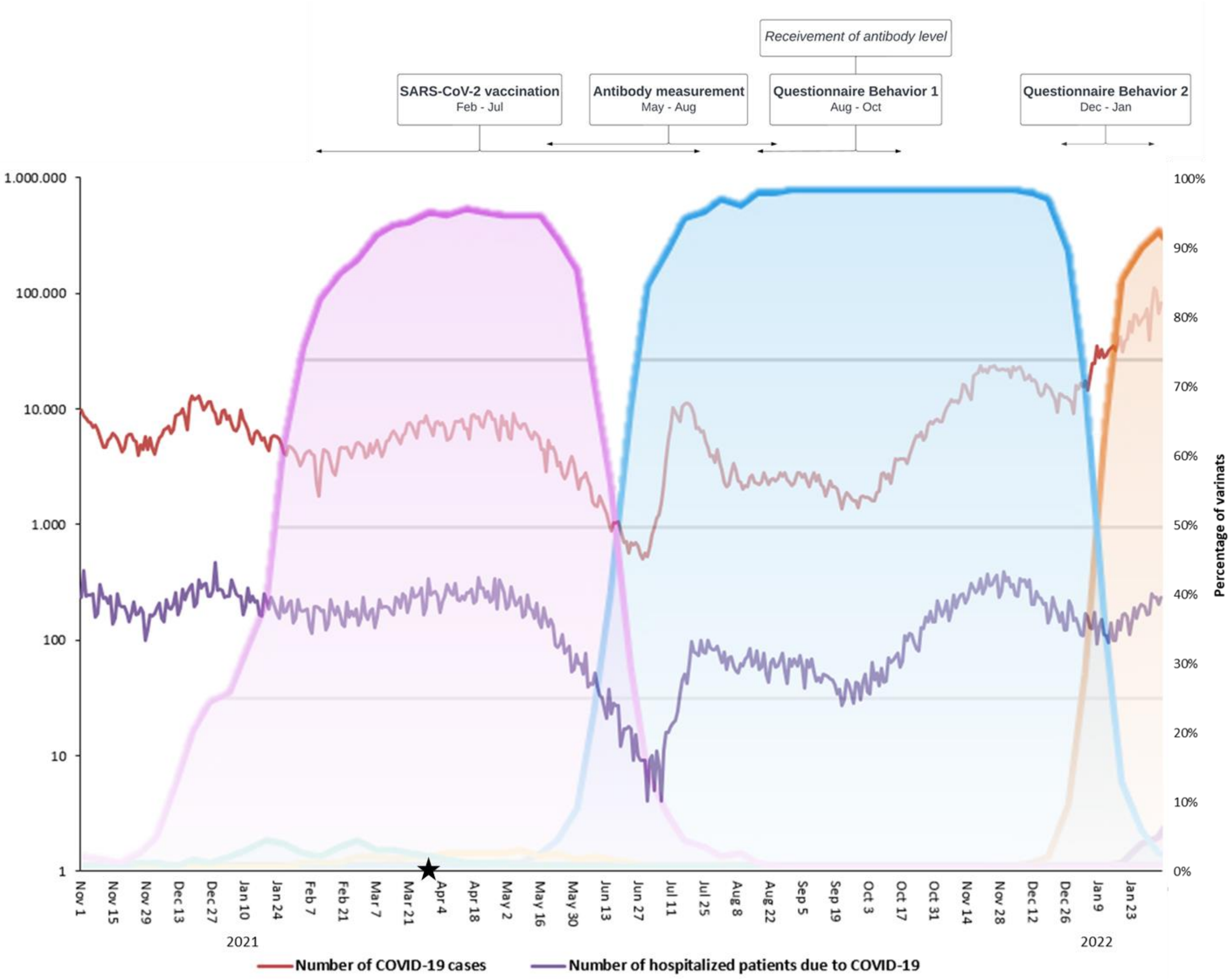

Figure S1. Overview of evolution COVID-19 pandemic during study period. Number of coronavirus disease 2019 (COVID-19) cases and hospitalized patients on log-scale according to date during the Alpha (purple), Delta (blue) and part of Omicron BA.1 wave (orange) in the Netherlands. The percentage of variants is displayed on the right axis (data from WHO COVID-19 Dashboard and National Institute for Public Health and the Environment (RIVM). The study timeline is stated on top. In Questionnaire Behavior 1 and 2 kidney transplant recipients (KTRs) reported their level of adherence to preventive measures before and after the second severe acute respiratory syndrome coronavirus-2 (SARS-CoV-2) vaccination respectively after awareness of antibody response. Time points of remaining events: information folder (April-June 2021) and two surveys containing questions about previous SARS-CoV-2 infection and general health (May-August 2021, December 2021-January 2022). The star symbol in the beginning of April 2021 represents the first availability of self-testing kits in the Netherlands.

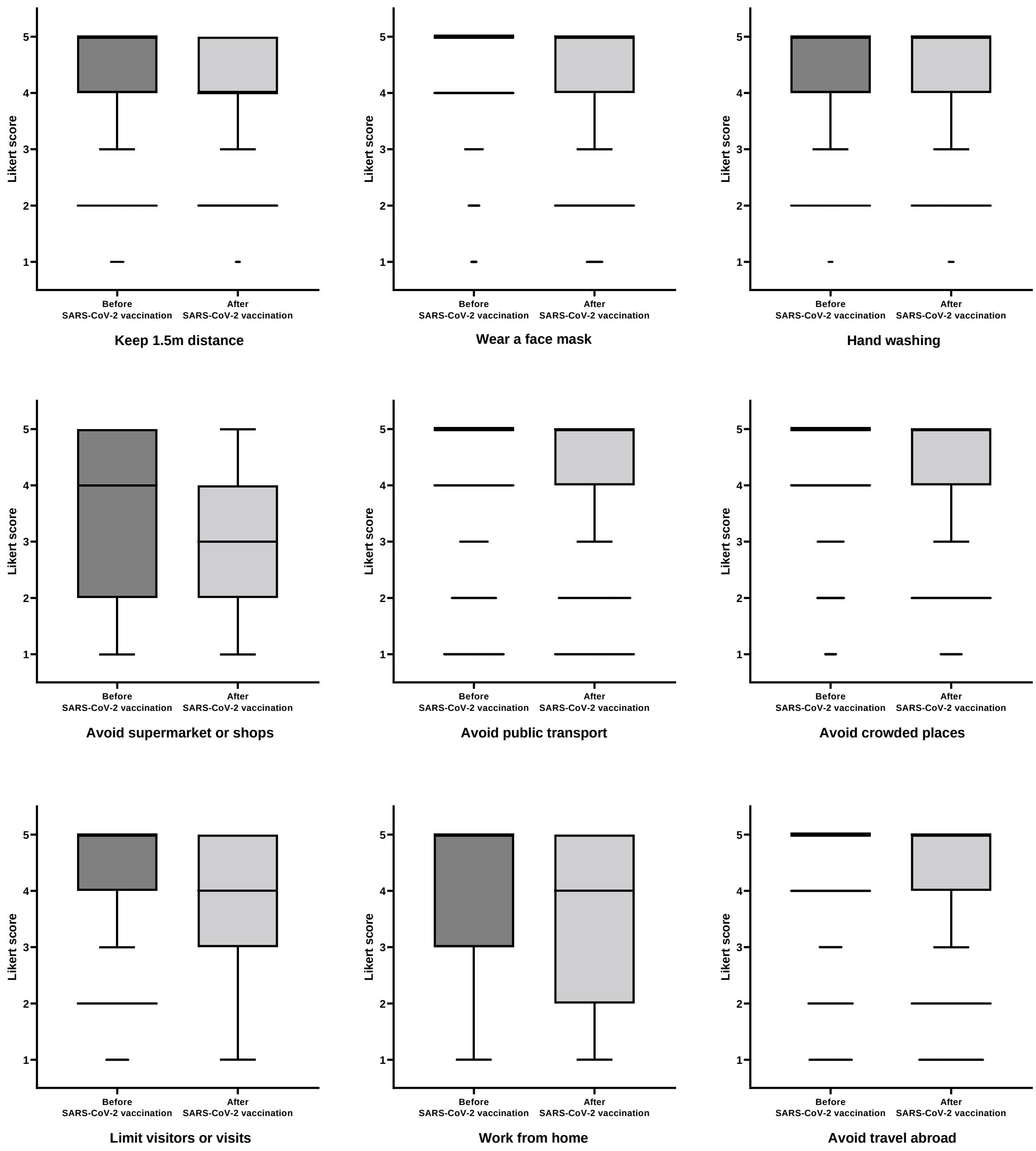

Figure S2. Boxplots for average adherence scores for each of the preventive measures before and after SARS-CoV-2 vaccination in KTRs (N=2939). Median and interquartile range (IQR) for average adherence scores for each of the preventive measures before respectively after severe acute respiratory syndrome coronavirus-2 (SARS-CoV-2) vaccination in kidney transplant recipients (KTRs) were as follows. ‘Keep 1.5m distance’ (5·00, IQR 4·00-5·00 and 4·00, IQR 4·00-5·00), ‘Wear a face mask’ (5·00, IQR 5·00-5·00 and 5·00, IQR 4·00-5·00), ‘Hand washing’ (5·00, IQR 4·00-5·00 and 5·00, IQR 4·00-5·00), ‘Avoid supermarket or shops’ (4·00, IQR 2·00-5·00 and 3·00, IQR 2·00-4·00), ‘Avoid public transport’ (5·00, IQR 5·00-5·00 and 5·00, IQR 4·00-5·00), ‘Avoiding crowded gatherings’ (5·00, IQR 5·00-5·00 and 5·00, IQR 4·00-5·00), ‘Limit visitors or visits’ (5·00, IQR 4·00-5·00 and 4·00, IQR 3·00-5·00), ‘Working from home’ (5·00, IQR 3·00-5·00 and 4·00, IQR 2·00-5·00), and ‘Avoiding travel abroad’ (5·00, IQR 5·00-5·00 and 5·00, IQR 4·00-5·00). Adherence was higher before than after SARS-CoV-2 vaccination for each of the preventive measures (p<·001).
