# Supplementary material for: Adherence to preventive measures after SARS-CoV-2 vaccination and after awareness of antibody response in kidney transplant recipients in the Netherlands: a nationwide questionnaire study
Source: eClinicalMedicine. 2023 Jul 20;62:102103. doi: 10.1016/j.eclinm.2023.102103 (PMC10393559; doi:10.1016/j.eclinm.2023.102103)
Supplement: Translated_abstract [file mmc3.docx]

The following translations in Dutch were submitted by the authors and we reproduce them as supplied. They have not been peer reviewed. Our editorial processes have only been applied to the original abstract in English, which should serve as reference for this manuscript.

**Samenvatting**

**Achtergrond** Niertransplantatiepatiënten (NTx) kregen het advies strikt de aanbevelingen vanuit de Overheid op te volgen om de verspreiding van ernstig acuut respiratoir syndroom coronavirus-2 (SARS-CoV-2) te beperken wegens het hoge risico op ziekte en overlijden en verminderde immunogeniciteit na vaccinatie. Het doel van deze studie was om de verandering in naleving van preventieve maatregelen na vaccinatie en na kennis van de antistofrespons te analyseren, en de effectiviteit ervan te evalueren.

**Methoden** In deze grootschalige, nationale vragenlijststudie werden vragenlijsten verstuurd naar 3531 NTx die deelnamen aan de Nederlandse RECOVAC-studies. Er werd retrospectief gevraagd naar de naleving van negen preventieve maatregelen op een 5-punts Likert schaal vóór en na SARS-CoV-2 vaccinatie, en na kennis van de antistofrespons. Bloedmonsters werden 28 dagen na de tweede vaccinatie verzameld. De antistofrespons werd gecategoriseerd als niet-responder (≤50 BAU/mL), laag-responder (>50 ≤300 BAU/mL) of hoog-responder (>300 BAU/mL), en gedeeld met de deelnemers als maat van bescherming. Deelnemers waarvan demografische gegevens over geslacht en leeftijd, bloedmonsters en ingevulde vragenlijsten beschikbaar waren, werden geïncludeerd. Onze studie vond plaats tussen februari 2021 en januari 2022. De primaire uitkomst van naleving vóór en na vaccinatie, uitgevraagd tussen augustus en oktober 2021, werd vergeleken met de Wilcoxon signed rank sum toets. Logistische regressieanalyse werd uitgevoerd om de associatie tussen antistofrespons en niet-naleving, en naleving en het oplopen van een SARS-CoV-2 infectie te schatten. Deze studie is geregistreerd bij ClinicalTrials.gov (NCT04841785).

**Resultaten** In 2939 NTx (83%) die de eerste vragenlijst over naleving van preventieve maatregelen hadden ingevuld, was de naleving hoger vóór dan na vaccinatie (4·56, IQR 4·11-4·78 en 4·22, IQR 3·67-4·67, p<0·001). Naleving na kennis van de antistofrespons werd geanalyseerd in 2399 NTx (82%) waarvan ook bloedmonsters beschikbaar waren, waarvan 949 niet-responders, 500 laag-responders en 950 hoog-responders. Vergeleken met niet-responders, rapporteerden laag- en hoog-responders een hogere niet-naleving. Een hogere naleving was geassocieerd met lagere infectiepercentages vóór en na vaccinatie (OR 0·67 [0·51-0·91], p=0·008 en OR 0·48 [0·28-0·86], p=0·010).

**Interpretatie** Het naleven van preventieve maatregelen nam af na SARS-CoV-2 vaccinatie en in NTx die kennis hadden van een daaropvolgende antistofrespons, in vergelijking met NTx zonder antistofrespons. Preventieve maatregelen in deze kwetsbare groep zijn effectief, ongeacht de vaccinatiestatus.

**Financiering** ZonMw en de Nederlandse Nierstichting.
